# Persistent Intraepithelial Lymphocytosis in Celiac Patients Adhering to Gluten-Free Diet Is Not Abolished Despite a Gluten Contamination Elimination Diet

**DOI:** 10.3390/nu8090525

**Published:** 2016-08-26

**Authors:** Barbara Zanini, Monica Marullo, Vincenzo Villanacci, Marianna Salemme, Francesco Lanzarotto, Chiara Ricci, Alberto Lanzini

**Affiliations:** 1 Gastroenterology Unit, University and Spedali Civili of Brescia, Piazzale Spedali Civili, Brescia I-25123, Italy; mon.ya@live.it (M.M.); francesco.lanzarotto@asst-spedalicivili.it (F.L.); chiara.ricci@unibs.it (C.R.); alberto.lanzini@unibs.it (A.L.); 2 Histopathology Unit, University and Spedali Civili of Brescia, Piazzale Spedali Civili, Brescia I-25123, Italy; villanac@alice.it (V.V.); mariannasalemme@alice.it (M.S.)

**Keywords:** gluten free diet, celiac disease, mucosal recovery, gluten contamination

## Abstract

The gluten-free diet (GFD) is the only validated treatment for celiac disease (CD), but despite strict adherence, complete mucosal recovery is rarely obtained. The aim of our study was to assess whether complete restitutio ad integrum could be achieved by adopting a restrictive diet (Gluten Contamination Elimination Diet, GCED) or may depend on time of exposure to GFD. Two cohorts of CD patients, with persisting Marsh II/Grade A lesion at duodenal biopsy after 12–18 months of GFD (early control) were identified. Patients in Cohort A were re-biopsied after a three-month GCED (GCED control) and patients in Cohort B were re-biopsied after a minimum of two years on a standard GFD subsequent to early control (late control). Ten patients in Cohort A and 19 in Cohort B completed the study protocol. There was no change in the classification of duodenal biopsies in both cohorts. The number of intraepithelial lymphocytes, TCRγδ+ (T-Cell Receptor gamma delta) T cell and eosinophils significantly decreased at GCED control (Cohort A) and at late control (Cohort B), compared to early control. Duodenal intraepithelial lymphocytosis persisting in CD patients during GFD is not eliminated by a GCED and is independent of the length of GFD. [NCT 02711696]

## 1. Introduction

Celiac disease is a systemic autoimmune disorder triggered by the ingestion of gluten, a complex of proteins contained in wheat, barley and rye, in genetically predisposed patients. The autoimmune process is responsible for alterations in the duodenal mucosa graded according to Marsh and the New Classification as Marsh I/Grade A (intraepithelial lymphocytosis), Marsh II/Grade A (+ glandular hyperplasia) and Marsh III (+ IIIa partial, IIIb subtotal or IIIc total villous atrophy)/Grade B (B1 low-moderate or B2 severe atrophy ) [1,2,3,4,5].

The only treatment for celiac disease is exclusion of gluten from the diet; however, despite strict dietary compliance, complete restitutio ad integrum of the duodenal mucosa is rarely achieved. In most cases, a variable degree of inflammation persists characterized by persistent intraepithelial lymphocytosis without or with associated glandular hyperplasia (Marsh I-II/Grade A) at control biopsy usually performed after 6–18 months of gluten-free diet (GFD) [6,7,8,9,10]. In our experience on 465 cases, complete normalization to Marsh 0 stage was observed in 8% of patients, with Marsh I and II lesions persisting in 65% of patients with duodenal atrophy at baseline [7].

The cause of persistence of this inflammation, even in patients fully adherent to GFD, poses a “million-dollar question” [11]. It is well known that there is a high degree of variability in individual response to gluten, with some patients’ worsening of duodenal histology on exposure to very small amounts of gluten [12,13,14]. This observation suggests that contamination with gluten from commercially available processed food and/or small amounts of gluten present in products labeled “gluten-free” (up to 20 ppm) may prevent complete mucosal recovery. This explanation is indirectly supported in a study by Hollon and colleagues [15] showing that persistence of gastrointestinal symptoms in celiac patients on a GFD is eliminated in 81% of cases by adopting a diet based exclusively on naturally gluten-free products, and on removal from the diet of commercially available processed food and products labeled “gluten free” (Gluten Contamination Elimination Diet, GCED). Unfortunately, information on the effects of this diet on duodenal histology was largely incomplete. Another explanation to gluten contamination is that complete mucosal recovery during GFD may go undetected, as the time of follow-up biopsies at 12–18 months may be too short.

The aim of our study was to assess whether complete recovery of duodenal mucosa in patients with persistent Marsh I-II/Grade A lesion after one year of GFD: (i) can be achieved by adopting a GCED; and/or (ii) may depend on the time of exposure to GFD. To achieve this aim, we studied two cohorts of patients with Marsh I-II/Grade A lesion after one year of GFD: cohort A was re-biopsied after three months on GCED, and cohort B after a minimum of two years subsequent to the first follow-up biopsy at 6–18 months.

## 2. Materials and methods

This study is a prospective, open label, interventional study involving celiac patients on regular follow up at our Celiac Disease Clinic during GFD. We identified 2 cohorts of patients.

### 2.1. Recruitment

Cohort A consisted of patients from the Clinic with persistent Marsh II/Grade A lesion at follow-up duodenal biopsy performed on GFD (early control), who were instructed to adopt a GCED for three months, before repeating biopsy. Cohort B consisted of patients on long-term follow-up, who accepted to repeat biopsy at least 60 months after the early control biopsy on GFD (late control). In both cohorts, patient selection criteria included: (i) Marsh II/Grade A lesion at duodenal biopsy performed at 12–18 months after starting GFD; (ii) negative CD related serology on GFD; (iii) strict adherence to GFD without digression; (iv) absence of gastrointestinal or extra intestinal symptoms; and (v) absence of *Helicobcter pylori* infection and no history of chronic NSAIDs (NonSteroidal Anti-Inflammatory Drugs) use.

Patients in cohort A were informed of the rationale for adopting a GCED and of the characteristics of the diet by a physician and a professional dietitian expert on CD and with the help of leaflets (AL and MM). Patients were seen monthly at the Clinic by the physician and the dietician to ensure adherence to the diet and to boost compliance with the protocol. The GCED was designed as indicated by Hollon et al. [15] and patients were required to stay on the GCED for a minimum of 3 months or more if well tolerated. Patients were given 2 kg of rice macaroni (Riso Viazzo, Crova, VC, Italy), 2 kg of rice (Riso Viazzo, Crova, VC, Italy) and 2 kg of corn flour (Molino Bresciano, Azzano Mella, Bs, Italy) free of charge for each month of the study. Before and after the GCED patients were asked to fill in a questionnaire on gastrointestinal symptoms using the Gastrointestinal Symptom Rating Scale (GSRS) questionnaire [16,17] that includes 15 items related to gastrointestinal symptoms grouped into 5 dimensions: abdominal pain, reflux, indigestion, diarrhea and constipation, and scored according to a 7-point Likert scale graded 1 (no discomfort at all) to 7 (very severe discomfort) referring to the previous week.

Patients in cohort B were assessed by the dietitian for adherence to GFD and invited to a repeated duodenal biopsy after long-term GFD (late control). Adherence to GFD was assessed by the dietician using 4 point Likert scale (1 = no digression; 4 = no adherence at all) as previously described [18].

### 2.2. Serology

IgA t-TG antibodies were measured on the same day as duodenal biopsy with the enzyme-linked immunosorbent assay procedure employing the human recombinant t-Tg antigen (Eu t-Tg® Eurospital, Trieste, Italy), and anti-endomysial antibodies (EMA) were detected by indirect immunofluorescence using monkey esophagus tissue as substrate (Antiendomysium®, Eurospital, Trieste, Italy). HLA genotype was assessed with commercial kits [7].

### 2.3. Histology

According to our Institutional protocol [7], a minimum of 4 endoscopic biopsies were obtained from the duodenum and specimens were oriented mucosal-up on cellulose filter. Duodenal histology was classified according to Marsh [1] and to the New Classification [3,4,5]. For pathology assessment two serials of 4-micron-thick were cut from each biopsy, one serial stained with H&E (Haematoxylin and Eosin) and one serial routinely used for CD 3 identification by immunohistochemistry using rabbit monoclonal antihuman antibodies 1:250 (Neomarker, Fremont, CA, USA). Intraepithelial lymphocytes (IEL) were counted on the H&E stained sections and on the corresponding CD 3 immuno-stained sections after counting at least 300 epithelial cells on both sides of five villous bodies. A cut-off value of 25 IEL/100 epithelial cells was used.

TCRγδ+ T cells were identified using a commercially available method suitable for formalin fixed paraffin embedded (FFPE) small bowel biopsies [19]. Serial cut sections (4 μm thick) of FFPE were stained using anti TCRCγM1 (Thermo Scientific, Fremont, CA, USA). After the appropriate antigen retrieval, the reaction was revealed using EnVisio (Dako, Glostrrup, Denmark) and Novolink polymer (Novocastra, Newcastle upon Tyne, UK). Diamonobenzidine was used as chromogen and Meyer’s hematoxylin as counterstaining. Sections were digitalized with Aperio Scanscope CS (Nikon) and a mean of the count was obtained with a cut off value of 4/100 epithelial cells. Eosinophils have also been counted with a cut off value of 2/100 epithelial cells. For Villous Height-Crypt Depth ratio (VH:CD), a cut-off value of 2 was considered to categorize patients as having active celiac disease (VH:CD <2) or not (VH:CD ≥2) [20]. All endoscopic biopsies were reviewed by experienced pathologists (V.V. and M.S.).

### 2.4. Statistics

Results were expressed as mean ± SD, and differences were tested for statistical significance using paired t test or Wilcoxon matched pairs test as appropriate, after testing normality with D’Agostino and Pearson omnibus normality test. A p value of <0.05 was used to reject the null hypothesis. The GraphPad Prism 5 statistical package (GraphPad Software, La Jolla, CA, USA) was used for statistical analysis and graphs.

### 2.5. Ethics

Our Institutional ethical committee approved of the study protocol on 10 February 2014. ClinicaTrials.gov identifier was NCT02711696.

## 3. Results

### 3.1. Patients’ Characteristics

**Figure 1:**
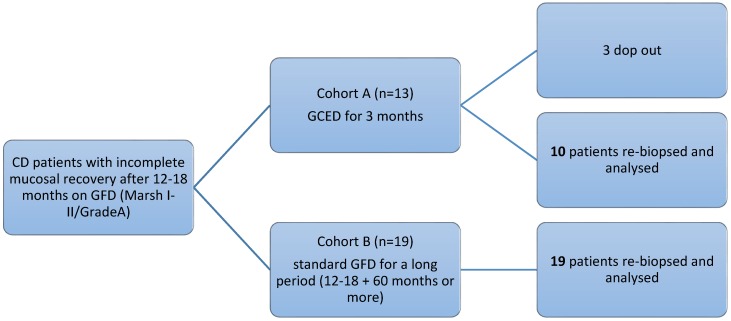
Study design flow-chart and patients assignment in the two cohorts.

Cohort A consisted of 13 patients (F 69%, mean age 38 ± 14 years) and cohort B of 19 patients (F 79%, mean age 34 ± 10 years). A flow chart of the study design and the main baseline characteristics in both cohorts reported in Figure 1 and Table 1, respectively.

### 3.2. Effect of GCED: Cohort A

Ten of the 13 patients enrolled in cohort A completed the trial. Two patients withdrew immediately after starting, and one patient after two months because of difficulties in adherence to GCED (patients 3 and 6, and 8 in Table 2, respectively).

As for selection criteria patients were virtually asymptomatic at control biopsy, and during GCED, GSRS dimension scores remained virtually unchanged for abdominal pain (2.40 ± 0.54 vs. 1.97 ± 0.81, *p* = 0.0954), reflux (1.7 ± 0.67 vs. 1.5 ± 0.85, *p* = 0.4962), diarrhea (2.23 ± 1.51 vs. 1.70 ± 0.97, *p* = 0.5736), constipation (2.03 ± 1.20 vs. 1.23 ± 0.35, *p* = 0.0502) and statistically improved for indigestion (2.93 ± 1.22 vs. 1.93 ± 0.83, *p* = 0.0183). All patients reported improvement in general wellbeing. There was no significant change in body weight with a tendency in patients to gain BMI (22.5 ± 5.2 vs. 23.0 ± 4.9, *p* = 0.5625).

**Figure 2:**
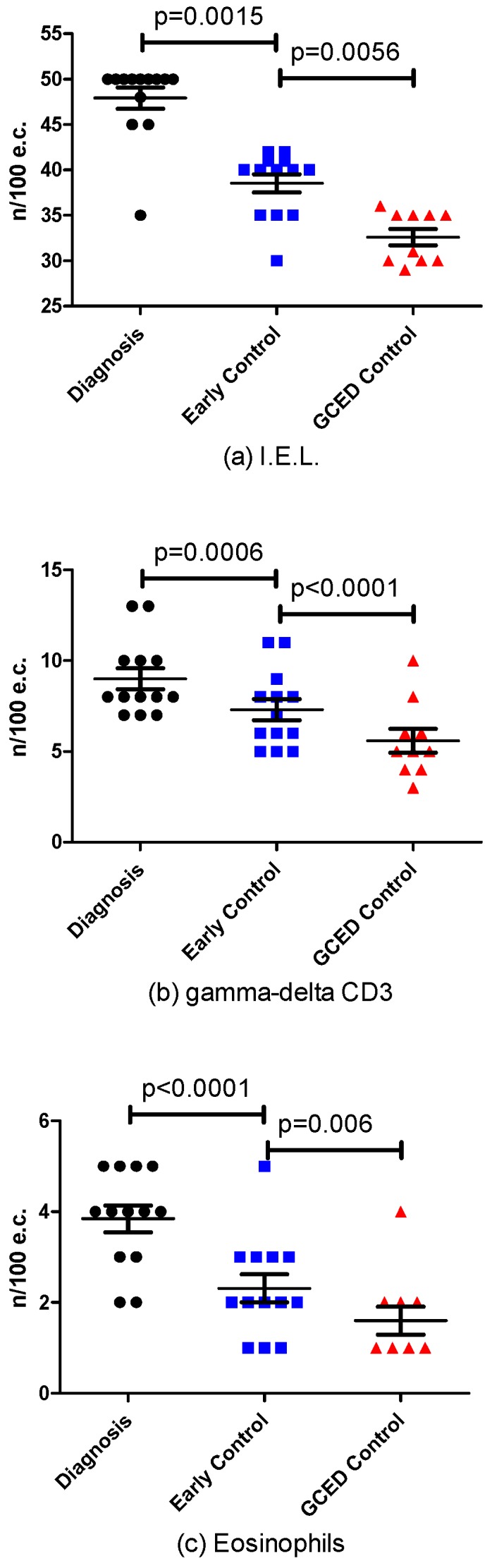
Histologic Changes at the time of diagnosis, at early control and during Gluten Elimination Contamination Diet (GCED) in the number of: IntraEpithelial Lymphocytes (IEL) (**a**); gamma-delta CD3 (**b**); and eosinophils (**c**).

There was no change in the classification of duodenal biopsies and in VH:CD category at the end of GECD compared with results of the early control biopsies taken after a mean 15 ± 4 months of GFD. In all patients, biopsies were classified as Marsh II/Grade A with VH:CD ≥2 on both occasions (Table 2). The number of intraepithelial lymphocytes decreased from 47.9 ± 4.3 at the time of CD diagnosis to 38.5 ± 3.6 at early control biopsies (*p* = 0.0015) with further decrease to 32.6 ± 2.8 after GCED (*p* = 0.0056). The number of TCRγδ+ T cells decreased from 9.0 ± 2.1 at the time of CD diagnosis to 7.3 ± 2.1 at early control biopsies (*p* = 0.0006) with further decrease to 5.6 ± 2.1 after GCED (*p* < 0.0001). The number of eosinophils decreased from 3.8 ± 1.1 at the time of CD diagnosis to 2.3 ± 1.1 at early control biopsies (*p* < 0.0001) with further decrease to 1.6 ± 1.0 after GCED (*p* = 0.006) (Figure 2).

### 3.3. Effect of Long Term GFD

**Figure 3:**
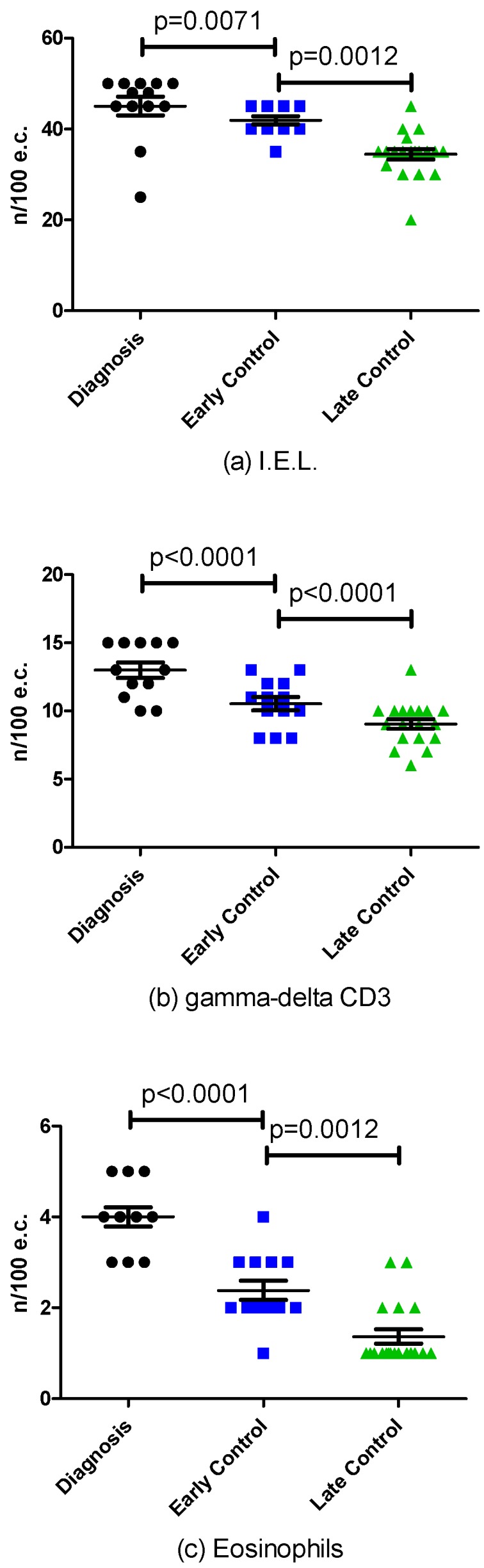
Histologic Changes at the time of diagnosis, at early and late control during Gluten Free Diet (GFD) in the number of: IntraEpithelial Lymphocytes (IEL (**a**); gamma-delta CD3 (**b**); and eosinophils (**c**).

Nineteen patients enrolled in cohort B had repeated biopsies 96 ± 47 months (range 54–216) after the first control biopsy taken 16 ± 7 months (range 12–36) after starting GFD (Table 3). There was no change in classification of duodenal biopsies and in VH:CD category after long-term GFD compared with results obtained at the early control biopsy. In all patients, biopsies were classified as Marsh II/Grade A with VH:CD ≥2 on both occasions (Table 3) and in no case revealed compete normalization. The number of intraepithelial lymphocytes decreased from 45.1 ± 7.3 at the time of CD diagnosis to 41.9 ± 3.3 at the early control biopsy (*p* = 0.0071) with a further decrease to 34.5 ± 5.1 after long-term GFD (*p* = 0.0012). The number of TCRγδ+ T cells decreased from 13.0 ± 2.0 at the time of CD diagnosis to 10.5 ± 1.8 at early control biopsy (*p* < 0.0001) with further decrease to 9.1 ± 1.5 after long-term GFD (*p* < 0.0001). The number of eosinophils decreased from 4.0 ± 0.7 at the time of CD diagnosis to 2.4 ± 0.8 at early control biopsies (*p* < 0.0001) with further decrease to 1.4 ± 0.7 after long-term GFD (*p* = 0.0012) (Figure 3).

## 4. Discussion

Our study shows that duodenal intraepithelial lymphocytosis persisting in celiac patients during GFD is not completely eliminated despite a GCED, and is independent of the time of adherence to GFD. Although the number of patients studied is limited, in none of the 10 patients completing the three-month GCED trial, there was a regression in the histological stage from Marsh II/Grade A to Marsh 0. Our finding, obtained in a prospective way, at predetermined time of histological assessment extends the similar retrospective observations obtained in four out of five patients studied by Hollon et al. [15]. Given these results, we felt it unethical to enroll more patients mainly because of the alarmed reaction elicited by the proposal of a trial dealing with “incomplete mucosal recovery“, and because adherence to the very restrictive GCED was very difficult, despite an improvement in wellbeing, mainly for social and psychological reasons, especially in asymptomatic patients.

The reason why GCED did not result in mucosal recovery is unclear. One possible explanation is that gluten contamination is unavoidable in our gluten-rich environment where the risk of contamination is high even in inherently gluten free food [21]. A Significant increase of IEL is an early event in the kinetics of histological response to gluten challenge in celiacs [22] thus acting as a sensitive marker of ongoing gluten ingestion. In our study, we did not allow for cereals except for rice and maize obtained by producers that exclusively process these two cereals. Besides contamination, a potential for maize prolamins to induce a gluten-like cellular immune response has been hypothesized [23] and this may contribute to persistent intraepithelial lymphocytosis at least in some patients.

A further possible explanation is that a three months of GCED is too short to achieve complete mucosal recovery and switching off the immunological process that characterizes CD. We have chosen a three-month period, because it proved sufficient in improving symptoms in 81% of the patients studied by Hollon et al. [15]. The study by Hollon et al. focused on the identification of refractory celiac disease (RCD) in non-responsive CD (biopsy-proven with persistence of symptoms and/or villous atrophy on strict GFD for at least 12 months) and on the proposal of a new algorithm to identify CD patients with RCD type 1 or type 2. Beside these differences in the study design, we felt unethical to ask our patients (asymptomatic and without villous atrophy on GFD) for a longer period of GCED.

A third possibility is that persistence of intraepithelial lymphocytosis during GFD and the more restricted GCED may be speculatively attributable to characteristics of intestinal microbiota. Intestinal microbiota has a well-recognized role in the shaping of the intestinal immune system [24,25] and research is focusing on its potential role in the pathogenesis of celiac disease as a key factor in association with genetic predisposition and gluten [26]. Many factors known to influence intestinal microbiota have been identified as risk factors for CD. These include caesarean delivery [27] and breast feeding practices [28], although evidence is conflicting [29,30,31], history of infection and antibiotics use [31]. Furthermore quantitative and qualitative differences of intestinal and fecal microbiota have been identified between CD patients and controls [32,33,34,35], and composition of duodenal microbiota appears to be in relation to clinical manifestations of CD [35]. An intestinal disbiosis also persists during GFD even after reconstitution of the villous structure [32,36,37] and is different in patients with persisting symptoms and those who are asymptomatic [38]. Small intestinal microbiota respond metabolically to dietary changes [37] and it is reasonable to hypothesize that dietary components of GFD and GCED may cause pro-inflammatory changes in the intestinal mucosa thus preventing the mucosa from progress beyond reconstitution of the villous structure. This hypothesis is supported in a recent study by Tjellstrom et al. [39] in children with CD showing that challenge with strictly gluten free oats caused an increase of in fecal SCFA, acetic acid and n-butyric acid consistent with changes in the gut microflora and resulting in increased “fermentation index”, a pro-inflammatory index.

Our study also confirms no further progression of intraepithelial lymphocytosis to normal limits after eight years of GFD compared with results obtained after 1–2 years [7,40]. Tuire et al. [40] reported a progressive reduction of in the prevalence of intraepithelial lymphocytosis from 85% at two years to 48% after 20 years of strict GFD and conclude that intraepithelial lymphocytosis may not disappear in treated CD over time. The reason for this persistence, besides common causes other than CD [41] that were excluded in our study and that of Tuire et al. [40] may, as in the case of lack of improvement with GCED, be related to gluten contamination or changes in microbiota during GFD.

Despite persistence of Marsh II/Grade A lesion, both patients treated with GCED and those on long-term GFD exhibited a slight although statistically significant reduction of IEL-CD3, Delta-gamma and eosinophils. This slight amelioration of the duodenal histology can be explained increased strictness in the GFD, that was inherent to the GCED, and the well established phenomenon of stricter adherence to dietary recommendations for patients enrolled in a clinical trial.

## 5. Conclusions

In conclusion, our study shows that residual duodenal inflammatory changes persist during GFD despite adherence to a diet based on products that are in nature gluten-free and despite long-term adherence to GFD. This incomplete mucosal recovery has, however, marginal clinical relevance and is unlikely to affect long-term prognosis [42].

## Figures and Tables

**Table 1:** Main characteristics of Cohorts A and B at the time of diagnosis of Celiac Disease.

Cohort A					Cohort B				
*n*	Gender	Age	Marsh	NC	*n*	Gender	Age	Marsh	NC
1	M	23	3a	B1	1	F	38	3c	B2
2	M	30	3c	B2	2	F	34	3c	B2
3	F	22	3c	B2	3	M	38	3c	B2
4	F	30	3a	B1	4	M	46	3c	B2
5	F	30	3a	B1	5	F	45	3c	B2
6	F	43	3b	B1	6	F	22	3a	B1
7	F	47	3b	B1	7	F	46	3a	B1
8	F	49	3c	B2	8	F	19	3	B1
9	F	26	3a	B1	9	F	25	3c	B2
10	F	68	3b	B1	10	F	42	3b	B1
11	M	44	3a	B1	11	F	20	3b	B1
12	F	29	3c	B2	12	F	44	2	A
13	M	58	2	A	13	M	32	3b	B1
					14	M	29	3b	B1
					15	F	26	3c	B2
					16	F	30	3c	B2
					17	F	26	3c	B2
					18	F	52	3b	B1
					19	F	38	3c	B2

NC: New Classification [3,4,5].

**Table 2:** Effect of Gluten Contamination Elimination Diet (GCED) on histological characteristics of duodenal biopsies.

Time of Diagnosis							Early Control (after 1 Year GFD)									GCED Control (after 3 Months of Restricted Diet)							
*n*	BMI	Ttg	VH/CD	IEL	γδ	Eos	Months	BMI	Ttg	Marsh	NC	VH/CD	IEL	γδ	Eos	Days	BMI	Marsh	NC	VH/CD	IEL	γδ	Eos
1	19.3	>100/16	<2	35	8	3	13	19.0	13/16	2	A	≥2	30	7	2	95	18.5	2	A	≥2	29	6	1
2	38.0	>100/16	<2	50	10	4	13	36.3	5.1/16	2	A	≥2	41	8	2	109	32.4	2	A	≥2	35	5	1
3	19.7	>100/16	<2	50	10	4	13	19.7	8.2/16	2	A	≥2	41	8	2	d.o.	-	-	-	-	-	-	-
4	18.6	7.5/7	<2	48	8	3	14	19.0	3.4/16	2	A	≥2	40	5	2	92	18.4		A	≥2	35	4	1
5	21.1	>100/16	<2	50	13	5	14	23.5	4.2/16	2	A	≥2	40	11	3	79	23.0	2	A	≥2	36	10	2
6	19.4	8.1/7	<2	50	13	5	16	20.0	5./16	2	A	≥2	40	11	3	d.o.	-	-	-	-	-	-	-
7	28.3	>100/16	<2	50	8	2	13	31.4	7.7/16	2	A	≥2	42	5	1	85	29.2	2	A	≥2	35	3	1
8	19.8	18/16	<2	50	8	2	15	19.2	5./16	2	A	≥2	42	5	1	d.o.	-	-	-	-	-	-	-
9	25.0	11.6/16	<2	45	10	5	15	24.6	3.2/16	2	A	≥2	35	8	3	109	24.1	2	A	≥2	30	6	2
10	20.0	29/16	<2	45	7	5	13	20.5	3.0/16	2	A	≥2	35	6	3	92	20.9	2	A	≥2	31	5	2
11	22.7	80/16	<2	50	8	4	14	22.3	4./16	2	A	≥2	40	6	2	95	21.7	2	A	≥2	30	5	1
12	19.3	>100/16	<2	50	7	4	15	19.7	7.9/16	2	A	≥2	40	6	1	99	20.4	2	A	≥2	35	4	1
13	22.9	8.8/16	<2	50	7	4	29	22.9	5.9/16	2	A	≥2	35	9	5	95	20.9	2	A	≥2	30	8	4

BMI: Body Mass Index; Ttg: Tissue Transglutaminases; NC: New Classification [3,4,5]; VH/CD: Villous Height/Crypt depth ratio; IEL: IntraEpithelial Lymphocytes; Eos: Eosinophils; d.o.: Drop Out.

**Table 3:** Effect of time on histological characteristics of duodenal biopsies.

Time of Diagnosis					Early Control (after 1 Year GFD)							Late Control (after Longer Period of GFD)						
*n*	VH/CD	IEL	γδ	Eos	Months	Marsh	NC	VH/CD	IEL	γδ	Eos	Months	Marsh	NC	VH/CD	IEL	γδ	Eos
1	<2	45	10	3	13	2	A	≥2	40	8	2	103	2	A	≥2	35	6	1
2	<2	50	15	4	12	2	A	≥2	45	12	3	54	2	A	≥2	35	10	2
3	<2	48	12	4	13	2	A	≥2	45	10	2	102	2	A	≥2	40	8	1
4	<2	48	13	5	16	2	A	≥2	45	11	3	78	2	A	≥2	40	10	1
5	<2	45	10	3	14	2	A	≥2	40	8	2	67	2	A	≥2	38	7	1
6	n.a	>25	n.a	n.a	12	2	A	n.a	>25	n.a	n.a	204	2	A	≥2	35	10	1
7	n.a	>25	n.a	n.a	12	2	A	≥2	45	10	4	54	1	A	≥2	35	8	3
8	<2	50	15	4	12	2	A	n.a	>25	n.a	n.a	216	2	A	≥2	45	13	1
9	<2	50	15	4	12	2	A	≥2	45	13	3	48	1	A	≥2	20	8	1
10	n.a	>25	n.a	n.a	12	2	A	≥2	40	11	2	63	2	A	≥2	35	9	1
11	n.a	>25	n.a	n.a	24	2	A	≥2	40	8	1	85	2	A	≥2	35	7	1
12	≥2	35	11	5	15	2	A	n.a	>25	n.a	n.a	80	2	A	≥2	32	9	3
13	n.a	>25	n.a	n.a	12	2	A	n.a	>25	n.a	n.a	60	2	A	≥2	35	9	2
14	<2	45	15	4	12	1	A	n.a	>25	n.a	n.a	63	2	A	≥2	30	10	1
15	<2	50	13	3	16	2	A	≥2	45	11	2	90	2	A	≥2	35	10	1
16	<2	50	12	5	13	2	A	≥2	40	10	3	116	2	A	≥2	35	9	2
17	n.a	>25	n.a	n.a	36	2	A	n.a	n.a	n.a	n.a	137	2	A	≥2	30	9	1
18	n.a	>25	n.a	n.a	22	2	A	≥2	35	13	2	85	2	A	≥2	30	10	1
19	<2	45	15	4	19	2	A	≥2	40	12	2	113	0	0	≥2	35	10	1

NC: New Classification [3,4,5]; VH/CD: Villous Height/Crypt Depth ratio; IEL: IntraEpithelial Lymphocytes; Eos: Eosinophils; n.a.: not available.
